# Conference of Ophthalmologists with Organization Officials of Michigan on Family Physician Refracting1Note by the Editor.—This report was sent to the Journal by Dr. A. A. Hubbell of Buffalo, with the request that it be published in this Magazine. Dr. Hubbell adds a foot note to the report expressing the opinion that it should be indorsed by the regents of the University of the State of New York. He says also that he has advocated for many years the restoration of all forms of medical practice, even the correction of errors of ocular refraction and motility, to the medical profession, where it belongs.

**Published:** 1910-08

**Authors:** Leartus Connor

**Affiliations:** Detroit, Mich. 91 Lafayette Boulevard


### Conference of Ophthalmologists with Organization Officials of Michigan on Family Physician Refracting[1]

By LEARTUS CONNOR, M.D.
Detroit, Mich.

THE promotion of family physician refracting calls for the coöperation of all physicians. It is believed that if these understood its value, all would seek its possession. To promote this understanding, the Detroit Ophthalmological Club invited to its meeting of May, 1910, the leading officials of Michigan medical societies and educational institutions. The following guests were present, took part in the discussions and endorsed the resolutions adopted—namely: Dr. J. H. Carstens, President Michigan State Medical Society; Dr. Winfred Haughey, Secretary-Editor Michigan State Medical Society; Dr. Frank B. Tibbals, Chairman Medico-Legal Committee, Michigan State Medical Society; Dr. Charles T. McClintock, Member of Committee on Organization, Michigan State Medical Society; Dr. Guy L. Kiefer, Health Officer of Detroit; Dr. W. C. Martindale, Superintendent Detroit Public Schools; Dr. Gerald Edmonds, of Honor, Mich., and Dr. W. C. Garvin, of Millington, Mich., family doctors doing simple refracting. The chairman of the council of the Michigan State Medical Society, Dr. W. T. Dodge, sent a letter supporting the movement. Though unable to be present, like support was given by the following gentlemen—namely, Dr. Victor C. Vaughan, Dean of the Medical Department of Michigan University; Dr. H. O. Walker, Secretary of the Detroit Medical College; Dr. Arthur D. Holmes, President of the Wayne County (Detroit) Medical Society.

The chairman of the meeting, Dr. Leartus Connor, opened the discussion by a paper on "The Economic Value of Family Physician Refracting." He called attention to the fact that in the United States were about 180,000,000 human eyes; 135,000 doctors; 3,000 ophthalmologists. This averages 60,000 eyes for each ophthalmologist. As every eye over forty years needs refracting, most of them several times, and as very many eyes under forty need refracting, we have an explanation of the fact that the ophthalmologists never have been able to meet the refractive needs of all the people in the United States. Because the family physicians never have been trained to supply these needs, salesmen of spectacles entered the field, and gave such

1. NOTE BY THE EDITOR.—This report was sent to the JOURNAL by Dr. A. A. Hubbell of Buffalo, with the request that it be published in this Magazine. Dr. Hubbell adds a foot note to the report expressing the opinion that it should be indorsed by the regents of the University of the State of New York. He says also that he has advocated for many years the restoration of all forms of medical practice, even the correction of errors of ocular refraction and motility, to the medical profession, where it belongs.

satisfaction as to secure large and increasing support. Further, without realizing the nature of their action, a large number of educated physicians support and patronise the opticians. In the hope of encouraging family physician refracting, the paper pointed out the vast values inherent in its universal adoption; values of ophthalmology and ophthalmologists, to general medicine and family doctor; to the profession as a whole, in strengthening its organisation; in solving the optician problem and in providing such coöperation of ophthalmologists with family doctor that the 180,000,000 human eyes in the United States may be cared for by educated physicians, so obviating the existing necessity for laymen ophthalmologists.

It was urged that if these values were accepted by this conference, the members could use their positions to stimulate others to comprehend the facts and master family physician refracting, till its enormous values be harvested for both profession and people. Simple refracting was shown to be an art quite within the reach of every medical student, without neglecting any other of his studies, and of practice by any doctor not already overburdened with work.

It was pointed out that while ophthalmology has many officers (specialists), it has few soldiers (family physicians) in its army, and so is impotent against the invasion of opticians with their horde of laymen. But an equipment of the entire profession with simple refracting would transfer all doctors to the ophthalmological army who, in turn, would capture the people, before opticians could reach them.

Every member of the club and its guests took part in the discussion. Some detailed the steps by which they had cast aside the dominant practice and adopted the one presented. Many virile reasons were adduced for the change, and suggestions made for promoting the new doctrine. Though lasting several hours, the conference never lagged but grew in interest from start to finish. Each saw in a new light a solution of present difficulties, vast benefits to the nation, and increased honor to the profession.

Without neglecting aught of present good work, members of the Detroit Ophthalmological Club and its guests of May 3d, 1910, adopted as their watchword "Family Physician Refracting" until every physician in the United States is equipped therefor.

Finally the following resolutions, on motion of Dr. Don M. Campbell, Professor of Ophthalmology in Detroit Medical College, were unanimously adopted by the Club:

WHEREAS, Family Physician Refracting promotes the coöperation of family physician and ophthalmologist; provides physicians adequate for the people's refractive needs; enriches ophthalmology and ophthalmologists; general medicine and family doctor; strengthens medical organisation and solves the optician problem;

*Resolved,* That the Detroit Ophthalmological Club endorses the action of the Ophthalmic Section American Medical Association, in promoting "Family Physician Refracting;" the action of the Michigan State Medical Society in seeking to qualify its members to meet the people's refractive needs; the action of the Kentucky State Medical Society endorsing the Michigan idea of family physician refracting; the action of the medical colleges in substituting "Family Physician Ophthalmology" for "Specialist Ophthalmology," and the action of the State Registration Boards in Michigan, Vermont, Nebraska and Utah in requiring a "working knowledge of simple refracting."

*Resolved,* That the Detroit Ophthalmic Club urge other State Boards of Registration to require (1) for license, "Family Physician Refracting" and (2) for the right to practise ophthalmolgy "a comprehensive laboratory and clinical study" after securing a license.

*Resolved,* That the Detroit Ophthalmic Club encourages family physicians to qualify for intelligent coöperation with ophthalmologists by equipping themselves with simple refracting.

It is suggested that much profit would result from other ophthalmic societies arranging similar conferences with officials of general or county medical societies, or even with groups of family doctors. Indeed, individual ophthalmologists might confer with little bands of family doctors and encourage them to master simple refraction, with the assurance of helpful results. Finally, family physicians doing simple refracting might so confer with neighbors and local societies, that all become more competent for the refractive needs of their families.

91 LAFAYETTE BOULEVARD.

